# P047: Fighting MRSA in an high endemic level hospital

**DOI:** 10.1186/2047-2994-2-S1-P47

**Published:** 2013-06-20

**Authors:** I Neves, V Alves, D Peres, F Vieira, I Devesa

**Affiliations:** 1 Infection Control Unit, Unidade Local de Saúde de Matosinhos., Matosinhos, Portugal

## Introduction

MRSA remains one of the principal resistant pathogens causing serious healthcare-associated and community-onset infections [1]. This agent is associated with increased morbidity, mortality risk and costs [2].

## Objectives

Monitoring and control of MRSA cases in a high endemic level scenario using multimodal strategy [3-5] in an 400 bed portuguese hospital.

## Methods

Multistep procedure involving isolation measures and active surveillance cultures (nasal swab using RT-PCR detection technique) in a selected population (patients from other hospitals and nursing homes; history of hospitalization/ MRSA; ICU patients and, in other inpatient services, direct contacts of newly detected MRSA patients). Since 2012, this cultures are also applied to patients doing hemodialysis. Other parallel activities: (a) review of isolation and standard precautions policy, (b) reinforcement of alcohol-based handrubs at point of patient care, (c) information sessions to health professionals, (d) targeted information flyer for health professionals, (e) information leaflet for patients/ visitors; (f) procedure monitoring by audit (g) patient decolonization in ICU, with follow-up screenings.

## Results

Between 2007 and 2012, MRSA surveillance detected a decrease in proportion from 66% to 57% and in density of incidence from 1.70 to 0,68 cases per thousand days of hospitalization. According to published data from EARSS, Portugal was the european country with the highest level of MRSA in 2011 [6]. In this network participated 22 portuguese hospitals and include 1507 isolates (blood and cerebrospinal fluid, only). Using this inclusion criteria, our Hospital reveled a proportion of MRSA below its national level (34% versus 55%) in 2011.

## Conclusion

Fighting MRSA using a multimodal strategy is being effective in a high endemic level hospital, but perseverance is needed through continuous surveillance of cases, feed-back to professionals and procedure audits.

## Disclosure of interest

None declared
